# Phytochemical analysis of green-branch bark extract and the brown gum exudates “kinos” from *Eucalyptus camaldulensis* by HPLC and GC–MS with their antifungal activity

**DOI:** 10.1038/s41598-026-38109-2

**Published:** 2026-02-23

**Authors:** Mohamed Z. M. Salem, Mohammed A. A. Elshaer, Abeer A. Mohamed, Mohamed A. M. Abd-Elraheem, Waled Abd-Elhamed, Tartil M. Emam

**Affiliations:** 1 Forestry and Wood Technology Department, Faculty of Agriculture (El-Shatby), Alexandria University, Alexandria, 21545 Egypt; 2 Agriculture Biochemistry Department, Faculty of Agriculture, Al-Azhar University, Sadat, Egypt; 3 Agriculture Research Center (ARC), Plant Pathology Research Institute, Alexandria, 21616 Egypt; 4 Agriculture Biochemistry Department, Faculty of Agriculture, Al-Azhar University, Cairo, 11823 Egypt; 5 Horticulture Department, Faculty of Agriculture, Ain Shams University, Cairo, Egypt

**Keywords:** Kinos, Eucalyptus camaldulensis, Antifungal activity, Phenolics, Flavoinods, Volatile compounds, Biochemistry, Biological techniques, Biotechnology, Drug discovery, Microbiology, Plant sciences

## Abstract

*Eucalyptus* has been utilized in traditional Australian medicines for the treatment of various ailments and is also used in pharmaceutical and cosmetic products. *Eucalyptus* contains an important source of key bioactive volatile and nonvolatile compounds. With the increasing research interest in *Eucalyptus* extracts and their health properties as an eco-friendly treatment, the green-branch bark extract (GBE) and the brown gum exudates, known as “kinos,” from *Eucalyptus camaldulensis* Dehnh. grown in Egypt, were used as biofungicide agents applied to *Pinus halepensis* Mill. wood samples. The phytochemicals were analyzed using the chromatographic tools, HPLC and GC–MS. These extracts at concentrations of 125, 250, 500, and 1000 µg/mL were further tested for their antifungal activity against *Fusarium circinatum* and *Pythium tardicrescens*, which were isolated from the diseased roots of *Pinus halepensis*. HPLC analysis of GBE revealed that kaempferol (14043.15 µg/g extract), gallic acid (7021.37 µg/g extract), and ellagic acid (4983.92 µg/g extract) were the major compounds. In the kinos, the main compounds were chlorogenic acid (12511.35 µg/g extract), gallic acid (12443.92 µg/g extract), ellagic acid (8147.54 µg/g extract), and rutin (2025.87 µg/g extract). By the GC–MS, *p*-cymene (31.91%), spathulenol (26.56%), and crypton (11.60%) were detected as primary compounds in the GBE. In the kinos, the abundant identified compounds by GC–MS were spathulenol (19.61%), isoaromadendrene epoxide (9.13%), *α*-acorenol (4.71%), and patchoulane (4.68%). Both GBE and kinos showed potential antifungal activity at 1000 µg/mL, inhibiting *F. circinatum* growth with fungal inhibition percentage (FIP) values of 71.85% and 71.11%, respectively. The GBE at 1000 and 500 µg/mL exhibited the highest antifungal effects against *P. tardicrescens*, with FIP values of 39.62% and 35.55%, respectively. The primary uniqueness of research into green-branch bark extracts and kinos from *Eucalyptus camaldulensis* comes from the growing global problem of antifungal resistance and the pressing need to identify specific bioactive chemicals for innovative development and investigate their application in environmentally friendly wood-biofungicide applications.

**Supplementary Information:**

The online version contains supplementary material available at 10.1038/s41598-026-38109-2.

## Introduction

*Eucalyptus camaldulensis* Dehnh. (Myrtaceae), commonly known as river red gum, is one of the most widely planted *Eucalyptus* species in arid and semi-arid regions, spanning over 90 countries, due to its exceptional drought tolerance and fast growth^1–4^. Only 5% of the tree is used for pool construction, and the majority of its industrial use (70–80%) is in the paper industry^5^, followed by carbon production (10–15%). This kind of industrial exploitation produces waste in the form of leaves and branches, which could potentially be a good source of bioactive compounds^4^. Beyond its agroforestry value, this species has long been utilized in traditional medicine—especially in Aboriginal Australian, North African, and South Asian practices—for treating wounds, gastrointestinal disorders, and microbial infections^6–8^. This tree is prized for its ecological relevance and therapeutic characteristics in addition to its timber and aesthetic appeal^9,10^. The food, pharmaceutical, and cosmetic industries have been particularly concerned with the chemical composition and biological properties of *Eucalyptus* extracts and essential oils^11,12^.

Two anatomically and biochemically distinct phytochemical reservoirs stand out: the green-branch bark, a lignified protective tissue, and the dark brown viscous exudates known as “kinos”, a defense secretion produced in response to injury or pathogen attack^4^. Despite ethnopharmacological prominence, comparative phytochemical and functional analyses of these matrices using modern hyphenated techniques remain underexplored, particularly concerning antifungal potential in the context of rising resistance to conventional agents^13^.

Numerous studies have examined the biological activities of bioactive chemicals isolated from the bark of *E. camaldulensis* against various pathogens, including bacteria, fungi, and viruses^3,7,14,15^. This activity is facilitated by the presence of phenolic compounds and essential oils, which make *Eucalyptus* bark a promising material for the production of natural antimicrobial agents. Several investigations were conducted on the utilization of *E. camaldulensis* extracts as natural antioxidants and anticancer agents^1,16,17^. The bark and leaves of *E. camaldulensis* have garnered attention in recent years due to their rich chemical composition, which includes a variety of bioactive compounds^18–21^.

Phenolic exudates or kinos often are mixed with terpenoid materials (the building block of exudates known as resins) and carbohydrates (the building block of exudates known as gums)^22^. Kinos from *Eucalyptus* trees are a trunk exudate that contains high levels of potentially useful polyphenols^23,24^.

Flavonoids have also been identified in kinos and the honey of *Eucalyptus* species^25,26^. They have played an important role in the traditional medicines of Australian Aboriginal people and were also a valued source of antibacterial and astringent agents for early European settlers^22,24^. All kino extracts from *Corymbia terminalis* in various solvents demonstrated bactericidal activity against some wound-associated bacterial strains, with the methanolic and crude aqueous extracts producing the largest inhibition zones^27^.

Phenolic and flavonoid compounds are the most bioactive chemicals in the extracts from *Eucalyptus*. A wide variety of polyphenolic compounds, such as hydrolyzable tannins, proanthocyanidins, flavanone glycosides, and formylated phloroglucinol compounds, were found in the extracts from the wound-associated wood that formed 17 months after artificial xylem injury in *Eucalyptus globulus* and *Eucalyptus nitens*^28^. There are significant quantities of flavonoids, with quercetin and catechin being the specific flavonoids identified in the extracts^1^. Gallic acid, *p*-hydroxybenzoic acid, syringic acid, and vanillic acid are among the phenolic acids that have been found, especially in polar extracts like the methanol extract^1^. Additional phenolic chemicals, such as kinotannic acid, catechol, and pyrocatechol, were revealed to be present in the kino exudates^4^. Saponins, flavonoids, tannins, and volatile oils were found in the crude methanol extract of *E. camaldulensis* stem bark from Bangladesh, but anthraquinones, hydrolyzable tannins, alkaloids, and glycosides were absent^29^. Candy that was made using a natural brown dye derived from eucalyptus bark was measured for its durability by assessing the amount of caffeic acid in the candies^30^.

*E. camaldulensis* is mostly used in the manufacturing of wood, paper, and charcoal; its leaves and branches are regarded as byproducts of these processes. Nonetheless, bioactive chemicals from these plant byproducts could be utilized to create new and enhanced consumer products^31^. Because of their substantial content of tannins, flavonoids, and phenolic compounds, which have strong antimicrobial, antioxidant, and anti-inflammatory properties, the green-branch bark and kinos (red gum exudate) from *E. camaldulensis* have a great deal of potential for a variety of applications. Therefore, to add potential significance for the uses of green-branch bark and kinos from *E. camaldulensis*, they were used as a wood-biofungicide to protect *Pinus halepensis* wood from the growth of some molds isolated from the same tree.

Pines are considered one of the most important components of natural forests, and the most commercially significant trees in the world, particularly in the Mediterranean region. Due to its adaptability, ease of cultivation, and lack of environmental requirements. Pine trees are susceptible to many diseases because their thin external tissues make them vulnerable to the entry of bacteria, viruses, nematodes, viroids, and fungi^32^. The pinewood fungus *Fusarium circinatum* and *Pythium tardicrescens*, which cause pitch canker and damping-off, respectively, are the most significant fungal infections^33,34^.

In light of this, the present study undertakes an integrated approach: detailed phytochemical profiling of *E. camaldulensis* green-branch bark and kino via orthogonal HPLC-DAD and GC–MS, with quantification of key markers, along with an evaluation of their antifungal activity when applied to wood samples.

## Materials and methods

### Preparation of extracts

This study has complied with relevant institutional, national, and international guidelines and legislation. This study does not contain any studies with human participants or animals performed by any of the authors. The green-branch bark and the brown gum exudates “kinos” from *Eucalyptus camaldulensis* (Fig. S1) were collected during 2025 from the tree growing at the nursery of Floriculture, Ornamental Horticulture and Garden Design, Faculty of Agriculture (El-Shatby), Alexandria University, Alexandria, Egypt. The plant was identified under the voucher number ME891 by Dr. Mervat EL-Hefny (Department of Floriculture, Ornamental Horticulture and Garden Design, Faculty of Agriculture (El-Shatby), Alexandria University, Alexandria, Egypt. The plant was further identified and deposited at the Herbarium of the Plant Production Department, Faculty of Agriculture (Saba Basha), Alexandria University, Alexandria, Egypt.

The gathered green-branch bark (GB) samples were air-dried at room temperature^35^. The dried bark was ground into a coarse particle size (20 mesh) using a small laboratory mill. Approximately 50 g of coarse particles of *E. camaldulensis* bark GB powder was macerated with 200 mL of methanol in a 2-L conical flask and shaken for 2 h. Then, it was left under laboratory conditions for three days to complete the extraction process. The mixture was then filtered using Whatman filter paper No. 1 to obtain the *E. camaldulensis* green-branch bark extract (GBE). The brown exudates (10 g) were dissolved in methanol (50 mL) and filtered through Whatman filter paper No. 1. The resultant kino extract was then dried by evaporating the methanol using a rotary evaporator at 60 °C. To complete the drying, the GBE was poured into Petri dishes under laboratory conditions.

### HPLC conditions for phytochemical analysis

The HPLC-DAD analysis of extracts from *E. camaldulensis* GB and kinos was carried out using an Agilent 1260 series device. The separation was performed using a Zorbax Eclipse Plus C8 column (4.6 mm × 250 mm, id, 5 μm film thickness). The mobile phase consisted of water (A) and 0.05% trifluoroacetic acid in acetonitrile (B) at a flow rate of 0.9 mL/min. A mobile phase linear gradient program was implemented with a step size of 1 min and durations of 5, 8, 12, 15, 16, and 20 min, using (A) at the concentrations of 82, 80, 60, 60, 82, 82, and 82%, respectively. The multi-wavelength detector was monitored at 280 nm. The injection volume was 5 µL for each sample solution (redissolved in acetone)^36^. The column temperature was maintained at 40 °C. Standard HPLC-grade phenolic and flavonoid compounds were used, including gallic acid, chlorogenic acid, catechin, methyl gallate, caffeic acid, syringic acid, pyrocatechol, rutin, ellagic acid, *p*-coumaric acid, vanillin, ferulic acid, naringenin, rosmarinic acid, daidzein, quercetin, cinnamic acid, kaempferol, and hesperetin. The identification of compounds was confirmed by comparing their retention time with the standard. All chemical standards (high-performance liquid chromatography (HPLC grade) were from Sigma‒Aldrich (St. Louis, MO, USA)^37^.

### Analysis of extracts by GC–MS

The possible chemical compounds that could be found in the GBE and kinos from *E. camaldulensis* were identified using Gas chromatography-mass spectrometry (GC–MS). A Trace GC-TSQ mass spectrometer (Thermo Scientific, Austin, TX, USA) with a direct capillary column TG–5MS (30 m × 0.25 mm × 0.25 μm film thickness) was used^38,39^. The column oven’s temperature was first kept at 50 °C, then increased by 5 °C/min to 250 °C for 2 min, and then increased by 30 °C/min to 300 °C for 2 min. The injector and MS transfer line were kept at 260 and 270 °C, respectively. Helium was used as a carrier gas at a constant flow rate of 1 mL/min. After a 2 min solvent delay, 1 µL diluted samples in *n*-hexane were automatically injected using the Autosampler AS1300 paired with GC in split mode. EI mass spectra were acquired at 70 eV ionization voltages in the m/z 50–650 range using full scan mode. The ion source’s temperature was set at 200 °C. The components were identified by matching their mass spectra to those of the NIST 14 and WILEY 09 mass spectral databases^40^. The Xcalibur 3.0 data system and threshold settings of the GC–MS were used to confirm that all of the mass spectra for the identified compounds were matched to the library. Additionally, the measurement match factor (MF) with values > 650 was used to confirm the identified chemicals^41^.

### Antifungal bioassay

The molecularly identified fungal isolates from the diseased root samples from *Pinus halepensis* (Mill.) (Fig. S2): *Fusarium circinatum* and *Pythium tardicrescens* (accession numbers PV636492 and PV636491), respectively^33^, were used for the antifungal activity. The prepared sapwood samples from *P. halepensis* with a dimension of 2 × 2 cm, with or without the concentrated extracts (125, 250, 500, and 1000 µg/mL) from the GBE, and kinos of *E. camaldulensis* were subjected to antifungal evaluation against the growth of two molds: *F. circinatum*, and *P. tardicrescens*. Each wood sample received 100 µL of the prepared concentrations of the extracts.

A week-old PDA medium was created for each type of fungus. One hundred microliters of the generated concentrations were given to each wood sample. In a Petri dish containing 15 mL of PDA medium, each fungal disc (5 mm in diameter) was introduced to the treated samples and controls. After that, the samples were grown at 28 °C for 7 days. Three duplicates of each fungus were utilized^42,43^. The inhibition percentage of fungal growth (IPFG) = [(Control growth - Growth in treatment)/Growth in control] × 100 was obtained and recorded for both treated and untreated woods against each fungus, using recommendations from previously published works^37,44^. The minimum inhibitory concentrations (MICs) of the GBE and kinos that were prepared at concentrations of 15.6 to 250 µg/mL were assessed using the broth dilution method according to CLSI^45^. Using the poisoned food approach, the positive control, Cure-M 72% WP (Mancozeb 64% + Metalaxyl 8%), was tested for antifungal activity at the suggested dosage (2 g/L) ^46^.

### Statistical analysis

The fungal inhibition percentages measured from the treated wood with the extracts were statistically analyzed. The statistical method was performed using two-way ANOVA (analysis of variance) in SAS software (SAS Institute, Release 8.02, Cary, North Carolina, USA), and the means were compared to the control treatment. The means of the treatments were compared using Duncan’s Multiple Range Test at the level of probability *p* < 0.05.

## Results

### Chemical compounds in the green-branch bark and kino extracts from *E. camaldulensis*

**Fig. 1:**
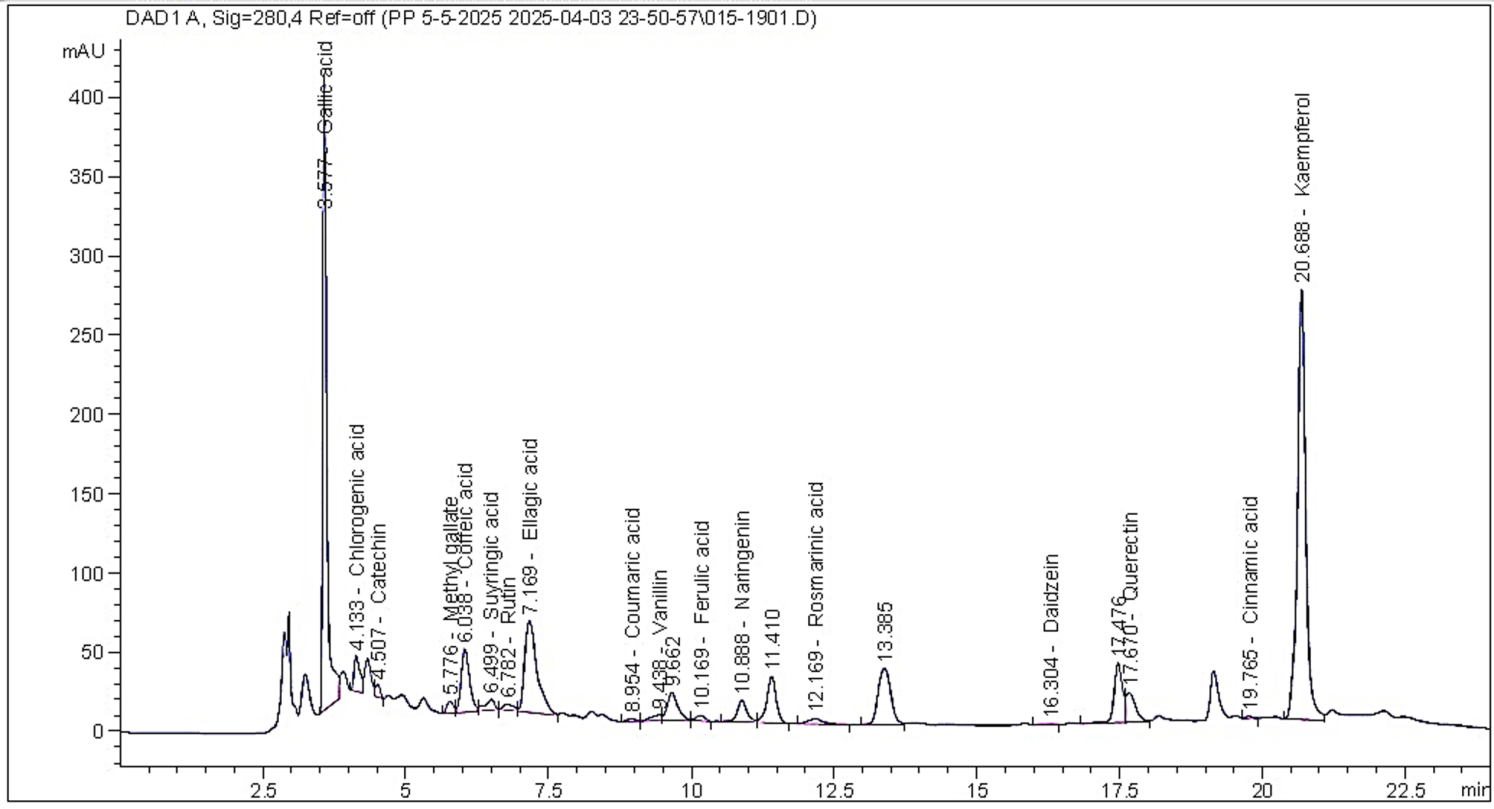
The HPLC chromatogram peaks of the identified compounds in the methanol extract from *Eucalyptus camaldulensis* green-branch bark extract.

The green-branch bark extract (GBE) was analyzed by HPLC analysis (Table 1; Fig. 1), where the main compounds were kaempferol (14043.15 µg/g extract), gallic acid (7021.37 µg/g extract), ellagic acid (4983.92 µg/g extract), quercetin (1447.17 µg/g extract), caffeic acid (1211.46 µg/g extract), chlorogenic acid (1143.84 µg/g extract), naringenin (799.73 µg/g extract), and catechin (546.32 µg/g extract).

**Fig. 2:**
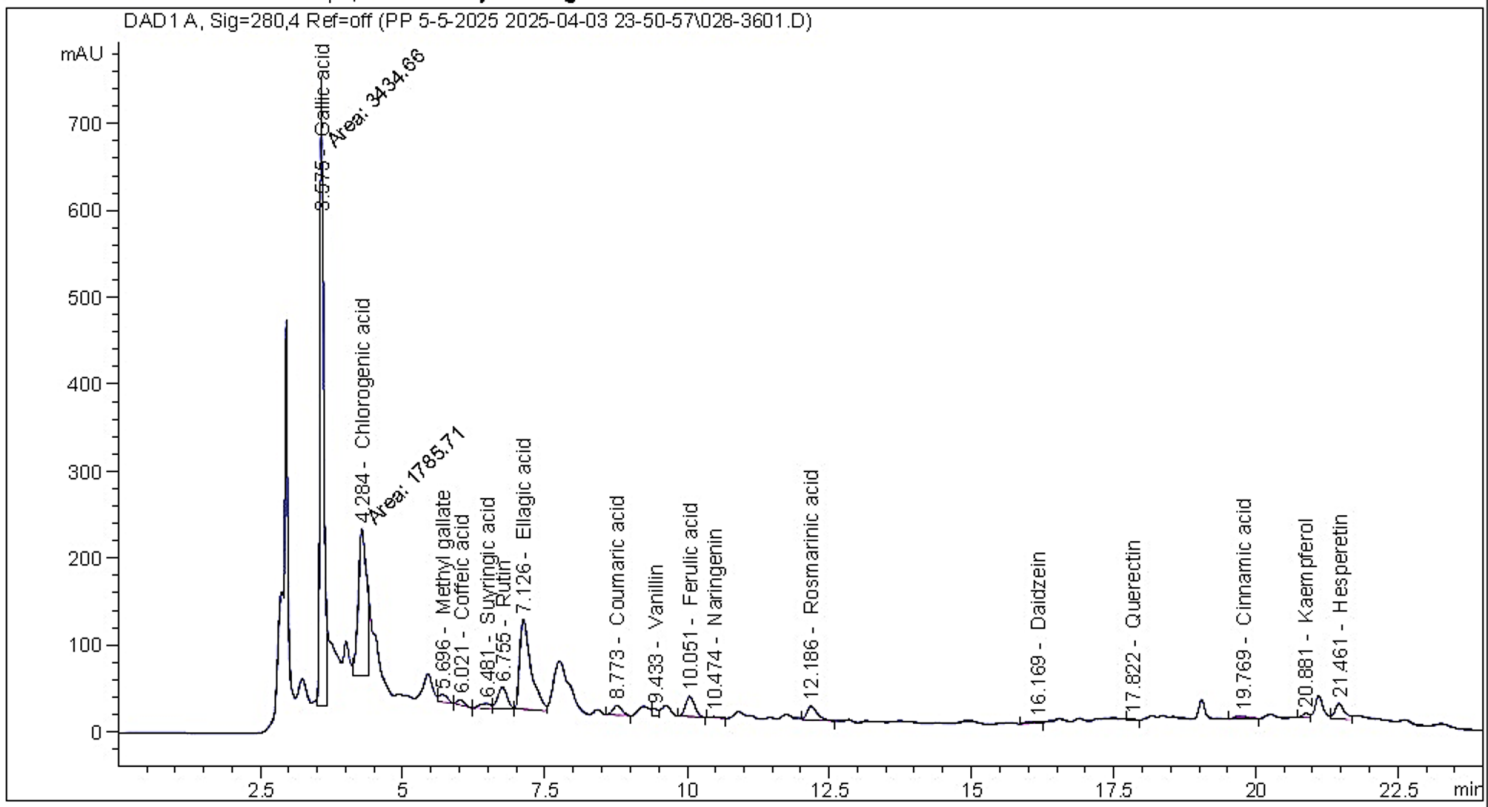
The HPLC chromatogram peaks of the identified compounds in *Eucalyptus camaldulensis* kinos.

The kinos material previously dissolved in the methanol solvent were analyzed by the HPLC analysis (Table 1; Fig. 2). The main compounds belong to phenolic and flavonoid types were chlorogenic acid (12511.35 µg/g extract), gallic acid (12443.92 µg/g extract), ellagic acid (8147.54 µg/g extract), rutin (2025.87 µg/g extract), rosmarinic acid (925.31 µg/g extract), ferulic acid (722.79 µg/g extract), and hesperetin (519.01 µg/g extract).

**Table 1:** The HPLC analysis extracts from *Eucalyptus camaldulensis*.

Compound	Green-branch bark extract					Kinos extract				
	RT*	Area [mAU*s]	Area (%)	Conc. (µg/mL extract)	Conc. (µg/g extract)	RT*	Area [mAU*s]	Area (%)	Conc. (µg/mL extract)	Conc. (µg/g extract)
Gallic acid	3.577	1937.98	23.011	140.43	7021.37	3.575	3434.66	42.21	248.88	12443.92
Chlorogenic acid	4.133	163.26	1.938	22.88	1143.84	4.284	1785.71	21.945	250.23	12511.35
Catechin	4.507	47.06	0.558	10.93	546.32	ND	ND	ND	ND	ND
Methyl gallate	5.776	61.24	0.727	3.47	173.58	5.696	86.09	1.06	4.88	244.04
Caffeic acid	6.038	404.74	4.805	24.23	1211.46	6.021	53.75	0.66	3.22	160.89
Syringic acid	6.499	81.02	0.962	5.19	259.33	6.481	74.61	0.92	4.78	238.83
Rutin	6.782	52.41	0.622	7.62	381.02	6.755	278.69	3.42	40.52	2025.87
Ellagic acid	7.169	876.68	10.409	99.68	4983.92	7.126	1433.17	17.61	162.95	8147.54
Coumaric acid	8.954	20.21	0.239	0.74	36.89	8.773	123.66	1.52	4.52	225.79
Vanillin	9.438	49.07	0.582	1.66	83.04	9.433	56.06	0.68	1.90	94.87
Ferulic acid	10.169	35.10	0.416	2.03	101.50	10.051	249.95	3.07	14.46	722.79
Naringenin	10.888	165.44	1.964	15.99	799.73	10.474	8.90	0.11	0.86	43.02
Rosmarinic acid	12.169	62.39	0.741	5.54	277.16	12.186	208.28	2.56	18.51	925.31
Daidzein	16.304	4.01	0.047	0.23	11.45	16.169	32.82	0.40	1.88	93.82
Quercetin	17.670	223.50	2.653	28.94	1447.17	17.822	11.89	0.15	1.54	77.00
Cinnamic acid	19.765	11.30	0.134	0.24	12.23	19.769	41.33	0.51	0.89	44.73
Kaempferol	20.688	2739.32	32.525	280.86	14043.15	20.881	38.08	0.47	3.90	195.20
Hesperetin	ND	ND	ND	ND	ND	21.461	219.51	2.69	10.38	519.01

*: RT: Retention time (min).

ND: Not detected.

**Fig. 3:**
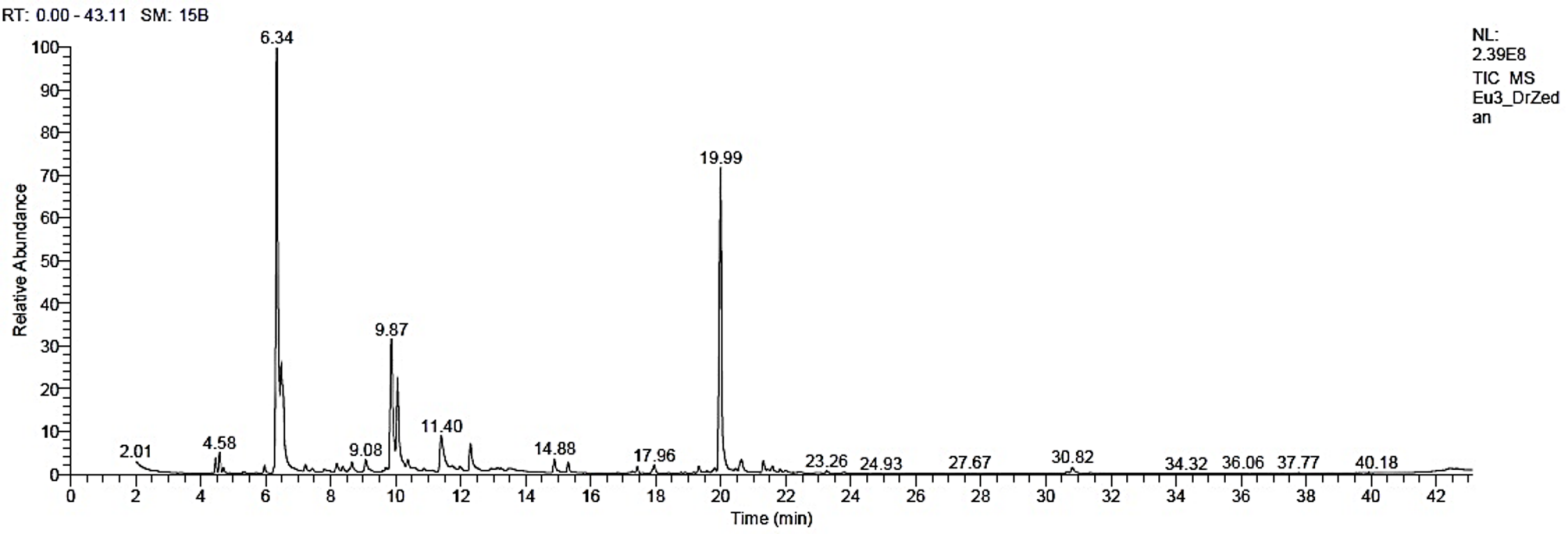
The GC–MS chromatographic peaks of the detected chemical compounds in the *Eucalyptus camaldulensis* green-branch bark extract.

The main chemical compounds found in the GBE (Table 2; Fig. 3) were *p*-cymene (31.91%), spathulenol (26.56%), crypton (11.60%), terpinen-4-ol (5.80%), cuminaldehyde (3.34%), eucalyptol (3.02%), D-limonene (2.31%), phellandral (2.26%), α-pinene (1.35%), *trans*-4-(isopropyl)-1-methylcyclohex-2-en-1-ol (1.06%), isospathulenol (1.05%), and *p*-(dimethoxymethyl)-isopropylbenzene (1.03%).

**Table 2:** The chemical compounds in the green-branch bark extract by GC–MS analysis.

RT^a^	Compound name	Area %	MF^b^	Molecular formula
4.46	3-Thujene	0.99	936	C_10_H_16_
4.58	α-Pinene	1.35	953	C_10_H_16_
4.71	2,4(10)-Thujadien	0.41	922	C_10_H_14_
5.96	2-Thujene	0.54	934	C_10_H_16_
6.35	*p*-Cymene	31.91	950	C_10_H_14_
6.48	Eucalyptol	3.02	838	C_10_H_18_O
6.54	D-Limonene	2.31	820	C_10_H_16_
7.22	*γ*-Terpinene	0.51	885	C_10_H_16_
8.18	Linalool	0.73	911	C_10_H_18_O
8.37	Thujone	0.37	906	C_10_H_16_O
8.65	trans-4-(Isopropyl)-1-methylcyclohex-2-en-1-ol	1.06	891	C_10_H_18_O
9.08	1-Terpinenol	0.98	799	C_10_H_18_O
9.87	Crypton	11.60	948	C_9_H_14_O
10.05	Terpinen-4-ol	5.80	895	C_10_H_18_O
11.39	Cuminaldehyde	3.34	964	C_10_H_12_O
12.29	Phellandral	2.26	952	C_10_H_16_O
14.88	*p*-(Dimethoxymethyl)-isopropylbenzene	1.03	974	C_12_H_18_O_2_
15.31	1-Cyclohexene-1-carboxylic acid, 2,6,6-trimethyl-, methyl ester	0.74	731	C_11_H_18_O_2_
17.43	Alloaromadendrene	0.45	948	C_15_H_24_
17.96	1,2,3,4-Tetrahydro-1,5-dimethyl-naphthalene	0.75	743	C_12_H_16_
19.32	α-Longipinene	0.51	862	C_15_H_24_
19.99	Spathulenol	26.56	949	C_15_H_24_O
21.31	Isospathulenol	1.05	867	C_15_H_24_O
21.58	Germacra-4(15),5,10(14)-trien-1a-ol	0.64	867	C_15_H_24_O
30.81	9-octadecenoic acid (Z)-, methyl ester	0.49	938	C_19_H_36_O_2_
Total identified compounds		99.4%		

^a^ RT; Retention time min.

^b^ MF: Match factor.

**Fig. 4:**
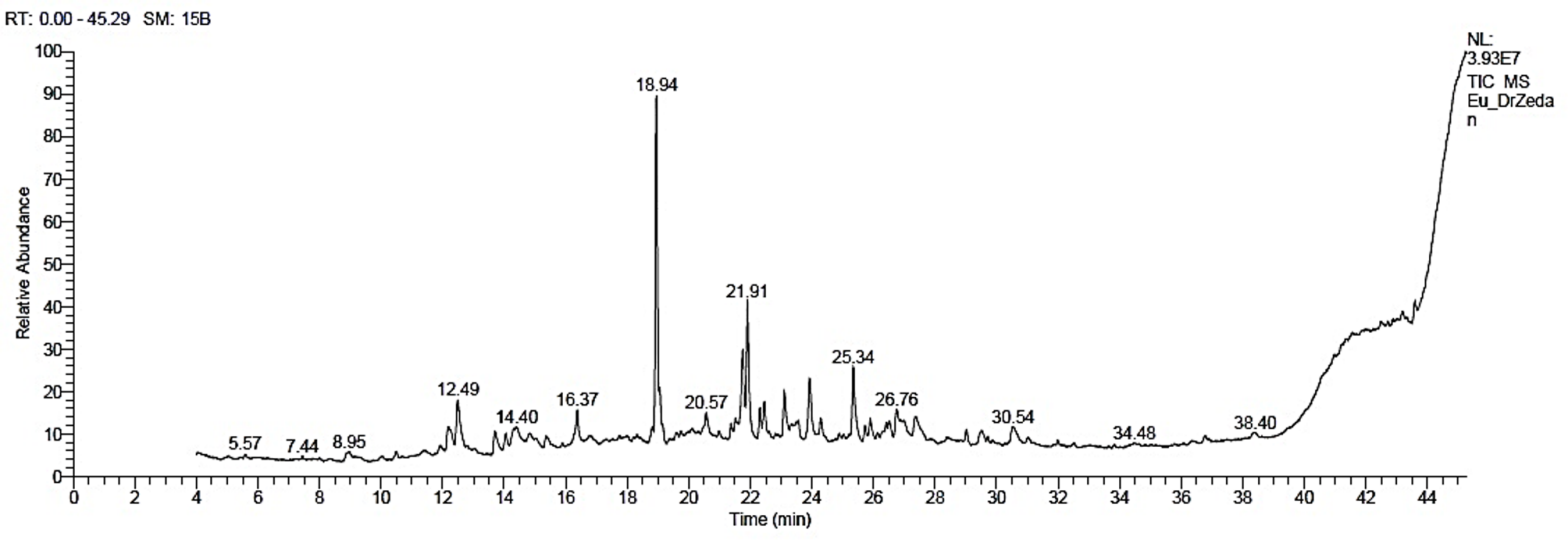
The GC–MS chromatographic peaks of the detected chemical compounds in the *Eucalyptus camaldulensis* kinos.

The main chemical compounds in the kinos from *Eucalyptus camaldulensis* by the GC–MS analysis (Table 3; Fig. 4) were spathulenol (19.61%), isoaromadendrene epoxide (9.13%), α-acorenol (4.71%), patchoulane (4.68%), methyl 5,7-hexadecadiynoate (3.71%), 3-ethyl-3-hydroxy-(5à)-androstan-17-one (3.56%), 6-methyl-cyclodec-5-enol (3.56%), doconexent (3.10%), 4-(2-methyl-3-oxocyclohexyl)butanal (2.81%), 7-hydroxyfarnesen (2.42%), epiglobulol (2.28%), aromadendrene oxide-(2) (2.06%), ledene oxide-(II) (1.95%), Z-(13,14-epoxy)tetradec-11-en-1-ol acetate (1.94%), 7-oxo-2-oxa-7-thiatricyclo[4.4.0.0(3,8)]decan-4-ol (1.82%), estra-1,3,5(10)-trien-17á-ol (1.81%), 6,9,12-octadecatrienoic acid methyl ester (1.55%), retinal (1.44%), docosa-6,9,12,15-tetraenoate (1.36%), 9,10-secochola-5,7,10(19)-trien-24-al, 3-hydroxy-, (3β,5Z,7E)- (1.29%), 2-[4-methyl-6-(2,6,6-trimethylcyclohex-1-enyl)hexa-1,3,5-trienyl]cyclohex-1-en-1-carboxaldehyde (1.25%), 2-(7-heptadecynyloxy)tetrahydro-2 H-pyran (1.23%), 2,2,4-trimethyl-4-(2-methyl-2-propenyl)hexahydrocyclopropa[cd]pentalene-1,3-dione (1.14%), undec-10-ynoic acid octadecyl ester (1.13%), 1-(cyclopropyl-nitro-methyl)-cyclopentanol (1.07%), tetrahydroactinidiolide (1.06%), and ascaridole epoxide (1.03%).

**Table 3:** The chemical compounds in the Kinos from *Eucalyptus camaldulensis* by GC–MS analysis.

RT^a^	Compound name	Area %	MF^b^	Molecular formula
5.58	1, 8-Cineol	0.35	701	C_10_H_18_O
7.44	3,6-Octadecadiynoic acid, methyl ester	0.21	795	C_19_H_30_O_2_
8.97	17-Octadecynoic acid	0.97	730	C_18_H_32_O_2_
10.49	R-Limonene	0.54	774	C_10_H_16_O_3_
11.39	*cis*-5,8,11,14,17-Eicosapentaenoic acid	0.99	720	C_20_H_30_O_2_
11.93	2-Ethylidene-6-methyl-3,5-heptadienal	0.70	731	C_10_H_14_O
12.18	4-(2-Methyl-3-oxocyclohexyl)butanal	2.81	754	C_11_H_18_O_2_
12.49	6-Methyl-cyclodec-5-enol	3.56	762	C_11_H_20_O
13.70	7-Oxo-2-oxa-7-thiatricyclo[4.4.0.0(3, 8)]decan-4-ol	1.82	818	C_8_H_12_O_3_S
14.05	1-(Cyclopropyl-nitro-methyl)-cyclope ntanol	1.07	767	C_9_H_15_NO_3_
14.31	Undec-10-ynoic acid, octadecyl ester	1.13	662	C_29_H_54_O_2_
14.39	Tetrahydroactinidiolide	1.06	726	C_11_H_18_O_2_
14.82	Octahydro-1,8a(1 h)-naphthalenediol	0.70	782	C_10_H_18_O_2_
15.36	Ascaridole epoxide	1.03	795	C_10_H_16_O_3_
6.37	Epiglobulol	2.28	796	C_15_H_26_O
16.80	(3β,5α)-Cholestan-3-ol, 2-methylene	0.82	791	C_28_H_48_O
18.02	Nona-2,3-dienoic acid, ethyl ester	0.35	756	C_11_H_18_O_2_
18.20	Arachidonic acid methyl ester	0.28	736	C_21_H_34_O_2_
18.31	9-(Acetyloxy)-3a,4,5,8,9,11a-hexahy dro-4-hydroxy-6,10-dimethyl-3-meth ylene-cyclodeca[b]furan-2(3 H)-one	0.55	712	C_17_H_22_O_5_
18.94	Spathulenol	19.61	947	C_15_H_24_O
19.05	Ledene oxide-(II)	1.95	829	C_15_H_24_O
19.38	Caryophyllene oxide	0.31	780	C_15_H_24_O
19.57	11,13-Dihydroxy-tetradec-5-ynoic acid, methyl ester	0.30	765	C_15_H_26_O_4_
19.63	Icosapentaenoic acid	0.22	748	C_20_H_30_O_2_
20.09	Methyl 4,7,10,13-hexadecatetraenoate	0.46	729	C_17_H_26_O_2_
20.56	Aromadendrene oxide-(2)	2.06	792	C_15_H_24_O
20.98	(E)-10-Heptadecen-8-ynoic acid methyl ester	0.48	760	C_18_H_30_O_2_
21.37	Retinal	1.44	878	C_20_H_28_O
21.51	11,13-Dihydroxy-tetradec-5-ynoic acid methyl ester	0.75	787	C_15_H_26_O_4_
21.74	α-Acorenol	4.71	801	C_15_H_26_O
21.91	Isoaromadendrene epoxide	9.13	947	C15H24O
22.45	7-Hydroxyfarnesen	2.42	865	C_15_H_24_O
22.82	(all-Z)-5,8,11,14-Eicosatetraenoic acid methyl ester	0.28	792	C_21_H_34_O_2_
23.10	Doconexent	3.10	772	C_22_H_32_O_2_
23.49	2 H-Pyran, 2-(7-heptadecynyloxy)tetrahydro-	0.35	733	C_22_H_40_O_2_
23.57	2,5-Octadecadiynoic acid methyl ester	0.70	799	C_19_H_30_O_2_
23.92	Methyl 5,7-hexadecadiynoate	3.71	794	C_17_H_26_O_2_
24.28	2,2,4-Trimethyl-4-(2-methyl-2-propenyl)hexahydrocyclopropa[cd]pentalene-1,3-dione	1.14	794	C_15_H_20_O_2_
24.89	1-Heptatriacotanol	0.38	782	C_37_H_76_O
25.34	Patchoulane	4.68	804	C_15_H_26_
25.72	cis-Z-α-Bisabolene epoxide	0.82	781	C_15_H_24_O
25.90	2-(7-Heptadecynyloxy)tetrahydro-2 H-pyran	1.23	751	C_22_H_40_O_2_
26.13	Pseudosolasodine diacetate	0.36	758	C_31_H_49_NO_4_
26.42	Nerolidol-epoxyacetate	0.87	826	C_17_H_28_O_4_
26.51	2-[4-Methyl-6-(2,6,6-trimethylcycloh ex-1-enyl)hexa-1,3,5-trienyl]cyclohe x-1-en-1-carboxaldehyde	1.25	760	C_23_H_32_O
26.75	3-Ethyl-3-hydroxy-(5α)-androstan-17-one	3.56	755	C_21_H_34_O_2_
26.99	Methyl docosa-6,9,12,15-tetraenoate	1.36	788	C_23_H_38_O_2_
27.35	Estra-1,3,5(10)-trien-17β-ol	1.81	776	C_18_H_24_O
28.37	cis-2-Phenyl-1,3-dioxolane-4-methyl Octadec-9, 12, 15-trienoate	0.46	728	C_28_H_40_O_4_
29.52	6,9,12-Octadecatrienoic acid, methyl ester	1.55	760	C_19_H_32_O_2_
29.71	4,9-Dihydroxy-6-methyl-3,10-dimethy lene-3a,4,7,8,9,10,11,11a-octahydro-3 H-cyclodeca[b]furan-2-one	0.36	757	C_15_H_20_O_4_
30.53	Z-(13,14-Epoxy)tetradec-11-en-1-ol acetate	1.94	782	C_16_H_28_O_3_
31.04	Ethyl iso-allocholate	0.45	763	C_26_H_44_O_5_
31.99	2-Hydroxy-1-(hydroxymethyl)ethyl ester (Z, Z,Z)-linolenic acid	0.36	728	C_21_H_36_O_4_
36.78	9,10-Secochola-5,7,10(19)-trien-24-a l, 3-hydroxy-, (3β,5Z,7E)-	1.29	794	C_15_H_20_O_5_
Total identified compounds		97.07%		

^a^ RT; Retention time min.

^b^ MF: Match factor.

## Antifungal activity

**Fig. 5:**
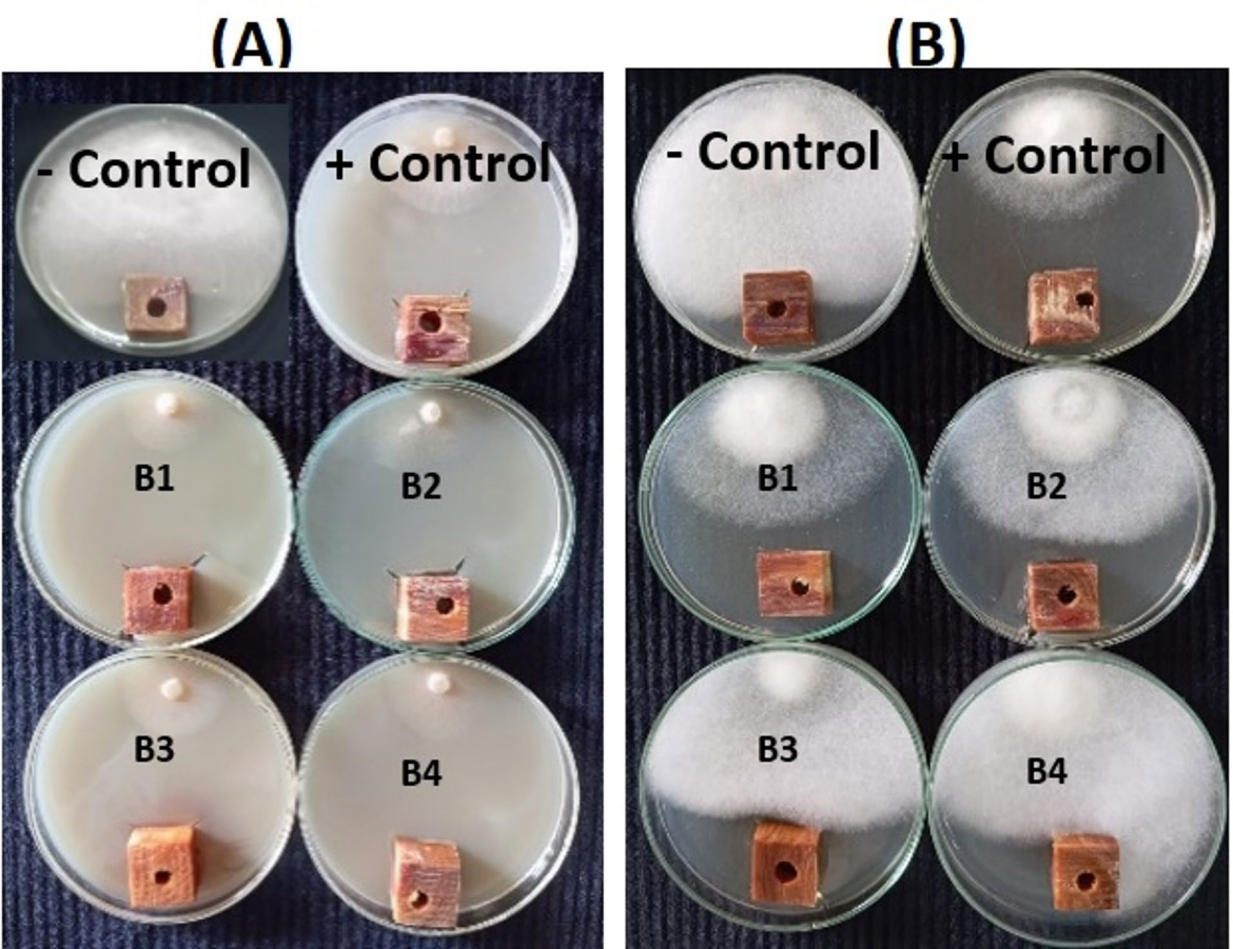
Visual observation of the antifungal activity of the green-branch bark extract when applied to *Pinus halepensis* wood against the growth of (**A**) *Fusarium circinatum*, and (**B**) *Pythium tardicrescens* at the concentrations of (B1) 1000 µg/mL, (B2) 500 µg/mL, (B3) 250 µg/mL, and (B4) 125 µg/mL.

**Fig. 6:**
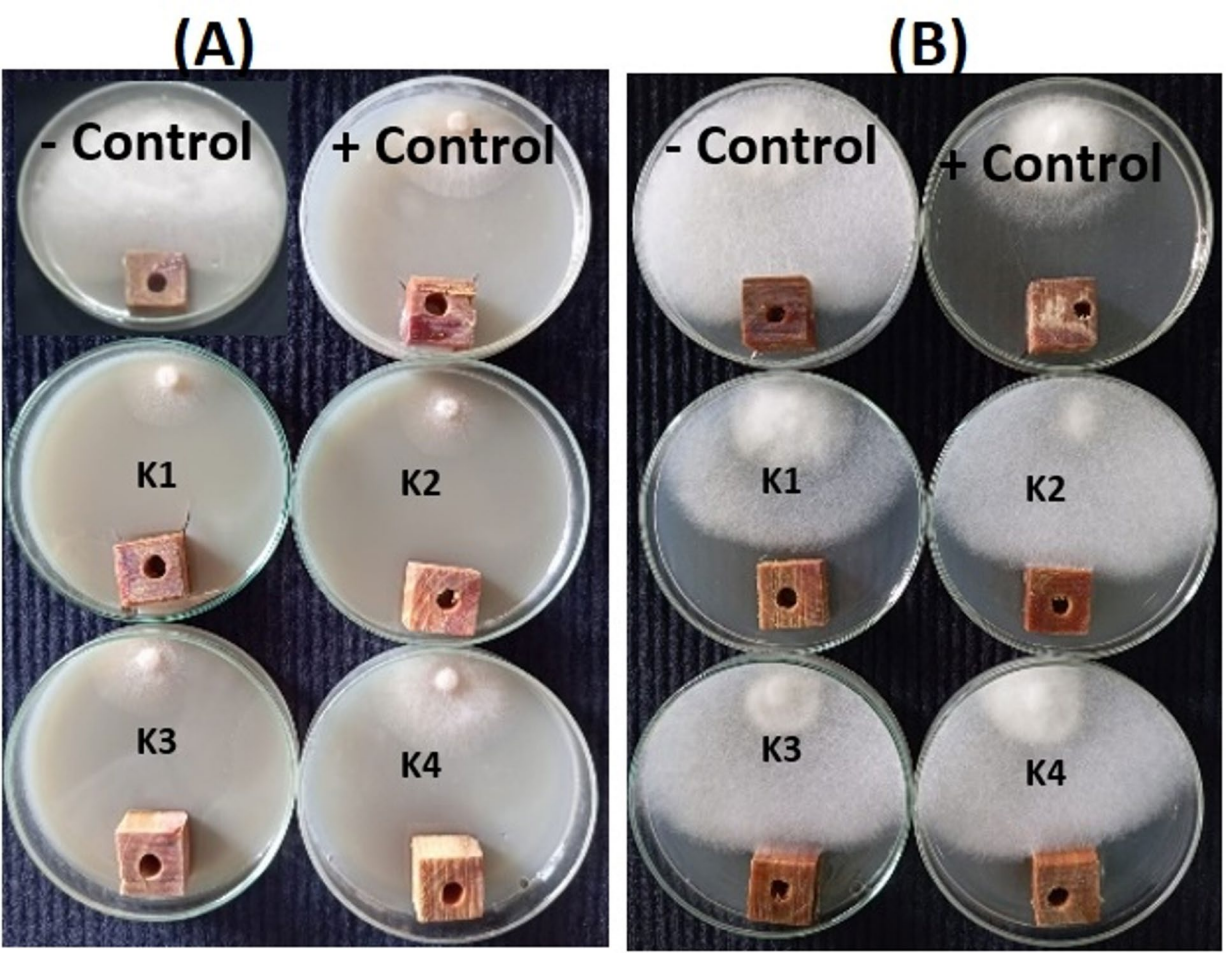
Visual observation of the antifungal activity of the kino extract when applied to *Pinus halepensis* wood against the growth of (**A**) *Fusarium circinatum*, and (**B**) *Pythium tardicrescens* at the concentrations of (K1) 1000 µg/mL, (K2) 500 µg/mL, (K3) 250 µg/mL, and (K4) 125 µg/mL.

Figures 5 and 6 show the visual observations of the antifungal activity of the GBE and kinos, respectively, when applied to *P. halepensis* wood against the growth of *F. circinatum*, and *P. tardicrescens* at the concentrations of 125, 250, 500, and 1000 µg/mL. These were compared to the negative control (10% DMSO) and the positive control, Cure-M 72% WP (Mancozeb 64%+Metalaxyl 8%), at the recommended dosage (2 g/L).

In Table 4, at a concentration of 1000 µg/mL, GBE and kinos demonstrated the highest activity against the growth of *F. circinatum*, with fungal inhibition percentage (FIP) values of 71.85% and 71.11%, respectively. These were followed by the kinos extract and GBE at 500 µg/mL, with FIP values of 66.66 and 66.29%, respectively. Additionally, GBE and kinos exhibited an FIP value of 61.11% at 250 µg/mL, which was higher than the value (FIP 58.88%) observed from the positive control (Cure-M 72% WP) at 2 g/L.

The GBE at 1000, 500, and 250 µg/mL showed the highest antifungal activity against the growth of *P. tardicrescens* with FIP values of 39.62, 35.55, and 32.96%, respectively. But these values are lower than those of the positive control (46.29%).

The minimum inhibitory concentrations (MICs) calculated for the treatments of *E. camaldulensis* GBE and kinos ranged between 15.6 and 62.5 µg/mL for both fungal isolates. The MIC calculated for the treatments of *E. camaldulensis* GBE was 15.6, and kinos was 31.3 µg/mL for *F. circinatum* isolate. The same result was observed with *E. camaldulensis* GBE and kinos. It was 62.5 µg/mL for *P. tardicrescens* isolate. The results showed that *E. camaldulensi*s GBE had a better effect on *F. circinatum* than on *P. tardicrescens* compared to kinos.

**Table 4:** Antifungal activity and the minimum inhibitory concentrations (MICs) of *Eucalyptus camaldulensis* green-branch bark and Kino extracts. Values are means ± SD; means with the same letter are not significantly different according to duncan’s multiple range test at the level of probability (*p* < 0.05). 1: positive control: Cure-M 72% WP (Mancozeb 64%+Metalaxyl 8%) at 2 g/L; 2: negative control: 10% DMSO.

Treatment	Concentration	Fungal inhibition percentage (FIP%)	
		Fusarium circinatum	Pythium tardicrescens
Negative control^1^	10%	0.00 g	0.00i
Positive control^2^	2 g/L	58.88d ± 0.00	46.29a ± 0.64
Green-branch bark extract	1000 µg/mL	71.85a ± 0.64	39.62b ± 0.64
	500 μg/mL	66.29b ± 0.64	35.55c ± 1.11
	250 μg/mL	61.11c ± 1.11	32.96d ± 0.64
	125 μg/mL	57.03e ± 0.64	30.74f ± 0.64
MIC	15.6–250 µg/mL	15.6 µg/mL	62.5 µg/mL
Kinos extract	1000 μg/mL	71.11a ± 1.11	31.85e ± 0.64
	500 μg/mL	66.66b ± 1.11	28.52 g ± 0.64
	250 μg/mL	61.11c ± 1.11	27.77 g ± 0.00
	125 μg/mL	54.07f ± 1.28	26.29 h ± 0.64
MIC	15.6–250 µg/mL	31.3 µg/mL	62.5 µg/mL
*p*-value		< 0.0001	0.0161

## Discussion

The chemical composition of *E. camaldulensis* bark is complex; however, its extracts are particularly rich in phenolic compounds, flavonoids, tannins, essential oils, and other secondary metabolites, which contribute to its antioxidant properties. The specific composition varies by factors such as clone, but major constituents include condensed tannins, phenolic acids (like gallic and syringic acids), and flavonoids (such as catechin). Polar extracts are also rich in polysaccharides, including glucans and xylans, while non-polar extracts can contain fatty acids like palmitic acid^47^. These compounds contribute to the tree’s defense mechanisms against pathogens and herbivores, as well as its adaptability to environmental stressors^48,49^.

In the present work, several phenolic and flavonoid compounds like gallic acid, chlorogenic acid, catechin, methyl gallate, caffeic acid, syringic acid, rutin, ellagic acid, coumaric acid, vanillin, ferulic acid, naringenin, rosmarinic acid, daidzein, quercetin, cinnamic acid, and kaempferol were detected in the green-branch bark (GBE) and kino extracts.

In *E. camaldulensis*, various phenolic acids, such as gallic acid, caffeic acid, and ferulic acid, have been identified^50,51^. These compounds display a variety of biological activities, including antimicrobial, anti-inflammatory, and anticancer effects. The level of phenolic compounds can change depending on environmental factors such as soil type, climate, and the age of the tree. The ethyl acetate fraction from leaf extracts led to the identification of six compounds: gallic acid, taxifolin, methyl gallate, quercetin, luteolin, and hesperidin^51^. Common flavonoids found in *Eucalyptus* bark include quercetin, kaempferol, and myricetin. The color of the bark and leaves, pollinator attraction, and UV protection are all influenced by flavonoids^48^. Numerous pharmacological effects, such as anti-inflammatory and anticancer properties, have been connected to their existence in *Eucalyptus* bark^52,53^.

Individual nonvolatile compounds have been isolated from various *Eucalyptus* species using GC and HPLC techniques, either alone or in combination with an auxiliary spectroscopic technique such as MS or NMR^54^. The phenolic compounds, such as gallic acid, protocatechuic acid, and ellagic acid^55,56^, have been identified in *Eucalyptus* extracts^57,58^.

The exudates extract analyzed by the HPLC showed the main compounds chlorogenic acid, gallic acid, ellagic acid, rutin, rosmarinic acid, ferulic acid, and hesperetin. Monomeric flavonoids and other phenolic chemicals have been found in kinos, with the bulk of them being intermediates that polymerize into tannins^59^. However, it is believed that these unique monomeric components are what give kinos their diverse physicochemical characteristics and, thus, their distinctive classification into the three “Maiden-groups” that were previously addressed.

Kinos from *E. largiflorens* showed the presence of methylated derivatives of gallic acid with other aromatic compounds, including 1,3,5-trimethoxybenzene and 1,3,5-trimethoxy-2-methyl-benzene^60^. Hydrolyzable tannins undergo methylation and hydrolysis to produce methylated derivatives of gallic acid and deoxy-sugar acids. The existence of varying methylation 3,6-deoxy-hexonic acid methyl esters in the pyrograms^61^, where gallic acid is connected to sugars via ester linkages, indicates the presence of sugars in kinos. Kaempferol was isolated from the kino of *Eucalyptus citriodora*^25^. Additionally, the narrow-leaved ironbark (*E. crebra*) honey identified tricetin, quercetin, luteolin, and kaempferol^26^. The wound-associated tissue of *E. nitens*, *E. globulus*, and *E. obliqua* contained a complex array of secondary metabolites, including hydrolyzable tannins, proanthocyanidins, flavonone glycosides, stilbene glycosides, formylated phloroglucinol compounds, volatile terpenes, and phenols^62^.

By the GC–MS analysis, several compounds, including *p*-cymene, spathulenol, crypton, terpinen-4-ol, cuminaldehyde, eucalyptol, D-limonene, phellandral, and α-pinene were found in the bark extract. Furthermore, the kino compounds analyzed by the GC–MS showed some bioactive compounds including spathulenol, isoaromadendrene epoxide, α-acorenol, patchoulane, methyl 5,7-hexadecadiynoate, 3-ethyl-3-hydroxy-(5à)-androstan-17-one, 6-methyl-cyclodec-5-enol, doconexent, 4-(2-methyl-3-oxocyclohexyl)butanal, 7-hydroxyfarnesen, epiglobulol, and aromadendrene oxide-(2).

GC–MS is essential for characterizing volatile and semi-volatile compounds, especially monoterpenes and sesquiterpenes linked to membrane disruption and efflux pump inhibition, in addition to polar metabolite identification^63–65^. *Eucalyptus* species are mainly known for their essential oils (EOs), though they can also be found in their bark. *E. camaldulensis* EO contains limonene, α-pinene, eucalyptol (1,8-cineole), spathulenol, and *p*-cymene^66–69^. These compounds, which give *Eucalyptus* its unique aroma, have been studied for their analgesic, antibacterial, and anti-inflammatory qualities. The sustainable approach of obtaining EOs from eucalyptus bark might be very beneficial to the fragrance and pharmaceutical industries. High concentrations of volatile organic compounds (VOCs) make up the EO profile of *Eucalyptus*^54^.

A precursor to carvacrol, *p*-cymene is a monoterpene with a benzene ring structure that can improve the cytoplasmic membrane’s permeability to adenosine triphosphate (ATP)^70,71^. It can enhance the antibacterial activity of other substances, in addition to exhibiting antimicrobial activity on its own. This is due to *p*-cymene’s strong affinity for microbial membranes and its ability to disrupt, expand, and influence the cell’s membrane potential^72,73^. *p*-Cymene has demonstrated significant antifungal properties, showing activity against various fungi, including *Aspergillus flavus*, *A. niger*, and *Fusarium culmorum*^74^. It often acts by disrupting cell membranes, inhibiting growth, and even working synergistically with other antifungal agents, such as miconazole^74,75^.

Spathulenol, with an MIC value of 100 µg/mL, was active against Citrus canker, which is caused by *Xanthomonas citri*^76^. The essential oil extracted from the *Hymenaea stigonocarpa* fruit peel, with its main compound spathulenol (25.19%), demonstrated antifungal activity against *Orytis cinerea*, *Sclerotinia sclerotiorum*, *Aspergillus flavus*, and *Colletotrichum truncatum*^77^. Spathulenol completely inhibited the formation of the fungal spores of *Aspergillus flavus*, *Fusarium culmorum*, and *Aspergillus niger* at a concentration of 50 µL/L over four natural fabrics (linen, cotton, wool, and silk)^21^. Potential action of spathulenol against several filamentous fungi and yeasts, including *Microsporum gypseum* and *Tricophyton mentagrophytes*, was noted^78^. *E. camaldulensis* EOs, and solvent-based extracts (leaf and bark) have been shown to have strong antifungal properties in numerous studies. For example, EOs show efficacy against a variety of fungi at doses of 0.125–1.0% (v/v), with *Fusarium sporotrichioides* being the most sensitive (MIC = 0.125%)^79^, and *Rhizopus oryzae* is the most resistant (no inhibition at 1.0%)^80^. Additionally, the methanolic leaf and bark extracts exhibited significant effectiveness against *Candida albicans* (MICs ranging from 0.2 mg/mL to 200 mg/mL)^81^.

Compounds isolated from the kinos of *C. citriodora*, such as 7-O-methylaromadendrin, 7-O-methylkaempferol, and ellagic acid, have demonstrated varying anti-fungal activities against the growth of *P. notatum*, *A. niger*, and *F. oxysporium*. Additionally, anti-bacterial activity against *Micrococcus pyogenes* var. *aureus* and *Mycobacterium phlei* has been reported in various fractions from the kino extract^84^. Bactericidal activity against *S. aureus* was demonstrated by the crude propolis made from the kino of *C. torelliana* and the extracted *C*-methyl flavones^85^. The antibacterial action of kino samples from *E. flocktoniae* and *E. sargentii* does not appear to be determined by the relative levels of hydrolyzable and condensed tannins^24^. High total phenolic and flavonoid content in *Grantia aucheri* extracts and the essential oils from *Cleome coluteoides* were responsible for the antifungal activity against pathogenic fungi, including *Candida albicans*, *C. glabrata*, *A. brasiliensis*, and *A. niger*^86,87^.

Wood and other natural materials were well protected by extracts, according to HPLC analysis for phenolic and flavonoid chemicals. When applied to model reference leather samples and produced cotton paper, *Pinus rigida* wood extract, which contains its primary constituents (cinnamic acid, caffeic acid, benzoic acid, quercetin, luteolin, and catechin), demonstrated strong antifungal activity against *A. flavus*, *A. niger*, and *Fusarium culmorum*^88^. With an increase in the extract concentration from leaves and branches of *Schotia brachypetala* when applied to white mulberry wood, the inhibition percentage against *Alternaria alternata*, *Botrytis cinerea*, and *Fusarium oxysporum* was increased. These were probably related to the presence of phytochemical compounds in the leaf extract, such as kaempferol and gallic acid, and gallic acid and chlorogenic acid in the branch extract^37^. Monoterpenes applied to *Pinus sylvestris* sapwood showed that *p*-cymene at 100 µL/mL had the highest fungal inhibition percentage against the growth of *A. flavus*; *p*-cymene and iso-eugenol against the growth of *A. niger*; and carvacrol against the growth of *F. culmorum*^89^. When applied to oak wood and *Imperata cylindrica* paper pulp, an aqueous extract of *Syzygium cumini* leaves, which contains the primary compounds benzoic acid, gallic acid, ellagic acid, and rutin, had some efficacy against *F. culmorum*, *A. fumigatus*, and *A. niger*^90^. Most recent work showed that the essential oil and recoverable extract from *Callistemon viminalis* leaves showed potential activity against the growth of *Fusarium culmorum*, *A. fumigatus*, and *A. niger* when applied to wood and linen, where the main compounds were pyrogallol and cinnamic acid^91^.

For the potential synergistic effects, when combined with traditional antibiotics (such as beta-lactams) and other plant extracts, the essential oil from *E. camaldulensis* extracts shows notable synergistic benefits that result in antibacterial efficacy, decreased drug resistance, and lowered required doses^92^. Using the paper disc diffusion method, the ethanolic leaf extracts of *E. camaldulensis* and *Psidium guajava*, as well as their combination, were found to have antibacterial properties in vitro against gram-positive *Staphylococcus aureus* and gram-negative *Escherichia coli*^93^. In Zimbabwe, a decoction of *E. camaldulensis* leaves was mixed with *Citrus limon* (L.) Burm. f. fruits and *Psidium guajava* L. leaves for fever, cough, and the flu; in Senegal, leaf decoctions were made with sugar for stomachaches^94,95^.

It has been demonstrated that spathulenol greatly increases the activity of common antifungals such as clotrimazole and fluconazole. Strong inhibitory effects are shown by extracts high in spathulenol against resistant strains of *Candida albicans*, *A. flavus*, and *A. niger*^96^. It functions as a biofungicide against plant-damaging fungi like *Botrytis cinerea* in addition to human diseases, frequently improving the efficacy of other synthetic or natural fungicides^97^.

According to studies, the minimum inhibitory concentration (MIC) of EOs and extracts from *E. camaldulensis* against fungi is typically much higher than that of common commercial antifungal medications like fluconazole or griseofulvin; however, because of their lower toxicity, they are regarded as a possible substitute^98^. When used as a positive control, standard fungicide (Apron star) demonstrated greater efficacy at lower concentrations (e.g., 15.00 mm inhibition zone for *F. solani* vs. 20.33 mm for undiluted EO, whereas *E. camaldulensis* EO demonstrated a MIC value of 7 to 8 µL/mL against *Fusarium* spp^98,99^. The EO against *Aspergillus flavus* and *Fusarium culmorum* had MICs of 8–40 µL/mL and 6–40 µL/mL, respectively, while Sertaconazole had MICs of 8 µL/mL and 6 µL/mL. Griseofulvin had a MIC of 0.064 mg/mL, but the leaf extract had a MIC of 6.4 mg/mL against *Trichophyton mentagrophytes*^8,100–102^.

One of the drawbacks of the study is the use of surfactants for the crude extract in practical formulations; further research and testing with different surfactants are needed. Several formulation studies can be used in the future to achieve this. It is important to keep in mind that a variety of conditions may affect the applied extract’s bioactivity, necessitating further investigation. Thus, further research into the long-term effects, or shelf life, of plant extracts when applied to the field of wood-biofungicides is made possible by this work.

Finally, the factors determining the cost of EOs and extracts from *E. camaldulensis* are several production-related factors, like plant yield, as *E. camaldulensis* is recognized for its high yields, and cultivation and harvesting of *E. camaldulensis*, as it is an evergreen tree, and all parts of the tree are rich in phytochemicals. Additionally, the method of extraction, such as steam distillation, is cost-effective for many plants, including *E. camaldulensis*, as well as using organic solvents for the extraction. Authentic, high-quality essential oils and extracts cost more. To guarantee purity and look for adulterants or synthetic fillers, which are frequently found in less expensive, inferior oils and extracts, reputable brands invest in stringent testing (such as GC–MS analysis). Transportation, packing in protective dark glass bottles, and a company’s overall business overhead all add to the final customer cost, making the supply chain and overhead another crucial component.

## Conclusion

Green branch-bark extract and kinos from *Eucalyptus camaldulensis* have a rich and varied chemical composition that includes a variety of bioactive substances with substantial potential for a range of uses. The tree’s ecological resilience and therapeutic qualities are attributed to the presence of phenolic, flavonoid, and volatile chemicals. When applied to wood samples of *Pinus halepinses*, both extracts showed possible effects against the growth of two molds, *Pythium tardicrescens* and *Fusarium circinatum*. The sustainable use of *Eucalyptus camaldulensis* bark extracts could support environmental preservation and economic growth, underscoring the significance of this unique species in the natural world.

## Supplementary Information

Below is the link to the electronic supplementary material.

Supplementary Material 1

## Data Availability

All data generated or analyzed during this study are included in this published article.
