# Supplementary material for: Phytochemical analysis of green-branch bark extract and the brown gum exudates “kinos” from Eucalyptus camaldulensis by HPLC and GC–MS with their antifungal activity
Source: Sci Rep. 2026 Feb 23;16:7480. doi: 10.1038/s41598-026-38109-2 (PMC12929632; doi:10.1038/s41598-026-38109-2)
Supplement: Supplementary file 1 — Supplementary Material 1 [file 41598_2026_38109_MOESM1_ESM.docx]

**Phytochemical analysis of green-branch bark extract and the brown gum exudates “kinos” from *Eucalyptus camaldulensis* by HPLC and GC-MS with their antifungal activity**

Mohamed Z. M. Salem ^a^, Mohammed A. A. Elshaer^b^, Abeer A. Mohamed^c^, Mohamed A. M. Abd-Elraheem^b^, Waled Abd-Elhamed^d^, Tartil M. Emam^e^

*^a^ Forestry and Wood Technology Department, Faculty of Agriculture (El-Shatby), Alexandria University, Alexandria 21545, Egypt;* [mohamed-salem@alexu.edu.eg](mailto:mohamed-salem@alexu.edu.eg)

*^b^* Agriculture Biochemistry Department, Faculty of Agriculture, Al-Azhar University, Sadat, Egypt; [mmm_elshaer@azhar.edu.eg](mailto:mmm_elshaer@azhar.edu.eg); [mohamedawad@azhar.edu.eg](mailto:mohamedawad@azhar.edu.eg)

*^c^ Plant Pathology Research Institute, Agriculture Research Center (ARC), Alexandria 21616, Egypt;* [abeera.mohamed81@gmail.com](mailto:abeera.mohamed81@gmail.com)

*^d^* Agriculture Biochemistry Department, Faculty of Agriculture, Al-Azhar University, Cairo 11823, Egypt; [waled.abdelhamed@azhar.edu.eg](mailto:waled.abdelhamed@azhar.edu.eg)

*^e^* Horticulture Department, Faculty of Agriculture, Ain Shams University, Cairo, Egypt; [tarteel_emam@agr.asu.edu.eg](mailto:tarteel_emam@agr.asu.edu.eg)

*: Corresponding author: [*mohamed-salem@alexu.edu.eg*](mailto:mohamed-salem@alexu.edu.eg)


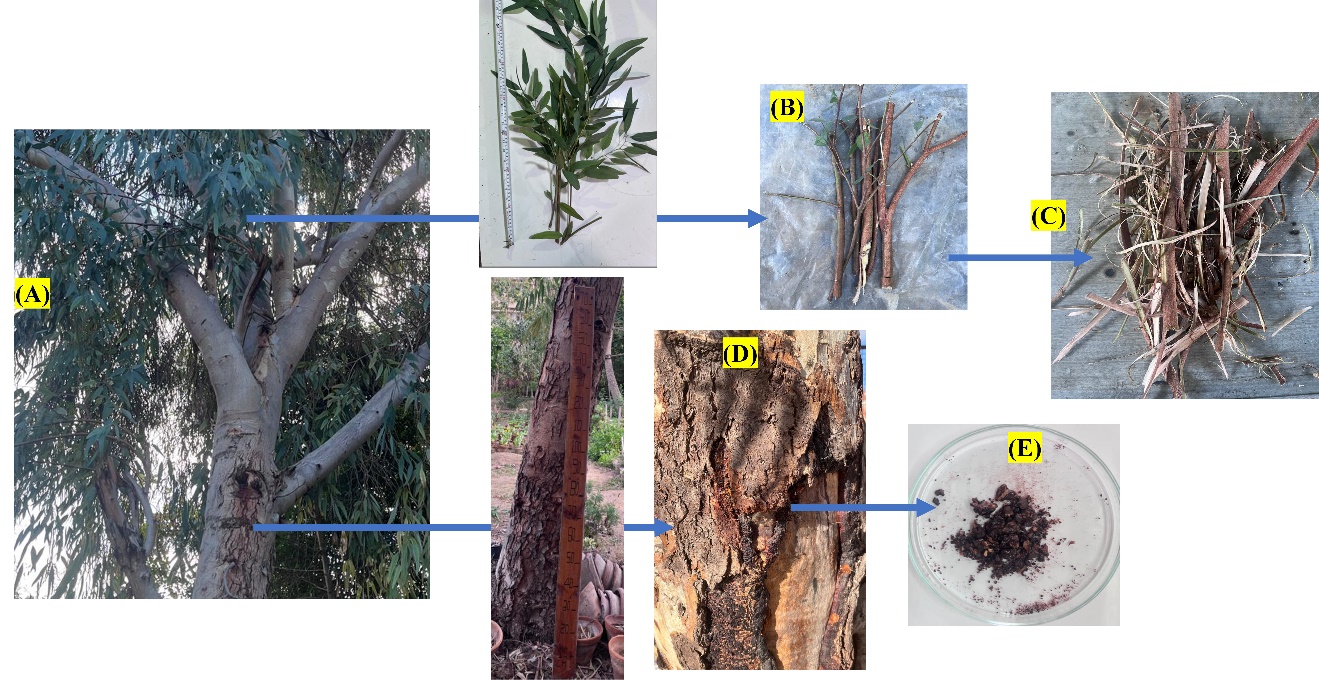


Fig. S1. *Eucalyptus camaldulensis* bark and kino. (A) *Eucalyptus camaldulensis* tree (the magnification 170 cm); (B) Branches (the magnification 60 cm); (C) green-branch bark; (D) Tree bark with the brown exudates (kinos); (E) the collected kinos.


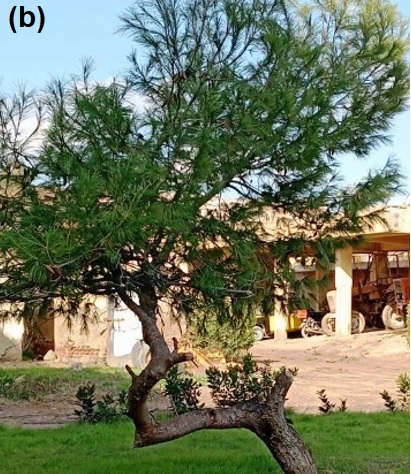


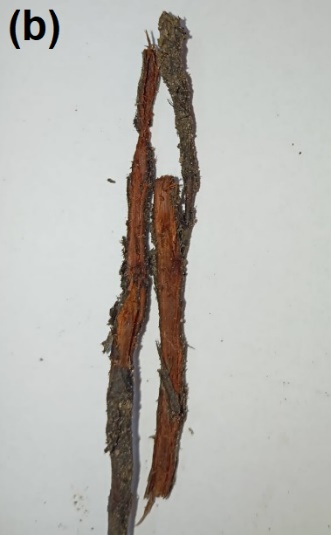


Figure S2. Photograph of the *Pinus halepensis* tree (a) and the diseased roots (b).
